# Body Fat Parameters, Glucose and Lipid Profiles, and Thyroid Hormone Levels in Schizophrenia Patients with or without Metabolic Syndrome

**DOI:** 10.3390/diagnostics10090683

**Published:** 2020-09-10

**Authors:** Elena G. Kornetova, Alexander N. Kornetov, Irina A. Mednova, Olga A. Lobacheva, Valeria I. Gerasimova, Viktoria V. Dubrovskaya, Ivan V. Tolmachev, Arkadiy V. Semke, Anton J. M. Loonen, Nikolay A. Bokhan, Svetlana A. Ivanova

**Affiliations:** 1 Mental Health Research Institute, Tomsk National Research Medical Center of the Russian Academy of Sciences, Aleutskaya str., 4, 634014 Tomsk, Russia; kornetova@sibmail.com (E.G.K.); irinka145@yandex.ru (I.A.M.); oalobacheva@mail.ru (O.A.L.); havssaltvg@gmail.com (V.I.G.); vika.dubrovskaya.vd@gmail.com (V.V.D.); semkeniipz@tnimc.ru (A.V.S.); bna909@gmail.com (N.A.B.); ivanovaniipz@gmail.com (S.A.I.); 2 University Hospital, Siberian State Medical University, Moskovsky trakt, 2, 634050 Tomsk, Russia; 3 Department of Fundamental Psychology and Behavioral Medicine, Siberian State Medical University, Moskovsky trakt, 2, 634050 Tomsk, Russia; alkornetov@gmail.com; 4 Department of Medical and Biological Cybernetics, Siberian State Medical University, Moskovsky trakt, 2, 634050 Tomsk, Russia; tolmachevivan82@gmail.com; 5 PharmacoTherapy, -Epidemiology and -Economics, Groningen Research Institute of Pharmacy, University of Groningen, Antonius Deusinglaan 1, 9713AV Groningen, The Netherlands; 6 Department of Psychiatry, Addictology and Psychotherapy, Siberian State Medical University, Moskovsky trakt, 2, 634050 Tomsk, Russia

**Keywords:** schizophrenia, metabolic syndrome, visceral fat, thyroid hormone, biochemical parameters, antipsychotics

## Abstract

In this study, we aim to investigate associations between body fat parameters, glucose and lipid profiles, thyroid-stimulating hormone (TSH), and thyroid hormones (THs) levels in Tomsk-region schizophrenia patients depending upon the presence or absence of metabolic syndrome (MetS). A total of 156 psychiatric inpatients with schizophrenia who had been treated with antipsychotics for at least six months before entry were studied: 56 with and 100 without MetS. Reference groups consisted of general hospital inpatients with MetS and without schizophrenia (*n* = 35) and healthy individuals (*n* = 35). Statistical analyses were performed using the Mann–Whitney U-test, chi-square test, Spearman’s rank correlation coefficient, multiple regression analyses, and descriptive statistics. Patients with schizophrenia and MetS had significantly higher levels of free triiodothyronine (FT3) and thyroxine (FT4) compared to schizophrenia patients without MetS (3.68 [3.25; 5.50] vs. 3.24 [2.81; 3.66], *p* = 0.0001, and 12.68 [10.73; 15.54] vs. 10.81 [9.76; 12.3], *p* = 0.0001, in pmol/L, respectively). FT3 maintained an association with MetS (*p* = 0.0001), sex (*p* = 0.0001), age (*p* = 0.022), and high-density lipoproteins (*p* = 0.033). FT4 maintained an association with MetS (*p* = 0.0001), sex (*p* = 0.001), age (*p* = 0.014), and glucose (*p* = 0.009). The data obtained showed body fat parameters, glucose and lipid profiles, and THs levels in Western-Siberian schizophrenia patients depending on MetS presence or absence.

## 1. Introduction

Schizophrenia is a multifactorial disorder and is considered a major burden for patients, their relatives, the health care system, and society. It contributes 13.4 (95% UI: 9.9–16.7) million years of life lived with a disability to the burden of disease globally [1]. The prevalence of schizophrenia in the general population is approximately 1%; rates across different countries, cultural groups, and sexes are similar. The disorder tends to begin between the ages of 16 and 30 years and mostly persists throughout the patient’s lifetime [2]. Schizophrenia is often accompanied by medical problems, such as autoimmune diseases, infections, chronic obstructive pulmonary disease, malignancies, and cardiovascular diseases. Somatic diseases are a major cause of the reduced life expectancy in schizophrenia patients, which amounts to 15–25 years less compared to that in the general population [3].

The mechanisms of the relationship between schizophrenia and physical illness are still not fully understood. On one hand, it is known that some atypical antipsychotics and poor lifestyle habits are associated with hyperglycemia, dyslipidemia, and weight gain, contributing to the overall risk of developing physical illnesses, including metabolic syndrome (MetS) and cardiovascular disease [4]. On the other hand, clinical studies have shown an increased risk for MetS in drug-naïve patients and first-degree relatives of people with schizophrenia [5], and treatment with antipsychotics is associated with lower mortality due to physical illnesses in patients with psychotic disorders [6]. The understanding that certain genes must affect multiple traits (a phenomenon termed pleiotropy) (because, in humans, millions of traits are regulated by only slightly more than 20,000 genes [7]) led Andreasen et al. [8] to combine the results of genome-wide association studies (GWASs) in a genetic-pleiotropy-informed procedure to investigate the relationship between schizophrenia and risk factors for cardiovascular disease.

The prevalence of MetS in people with schizophrenia is high [9]. Schizophrenia patients have a more frequent rate of MetS than that of individuals from the general population [10]. This appears true even though the MetS level varies in different countries due to differences in genetic origin, diet, physical activity, age and sex structure of the population, and body habitus. MetS is nevertheless a global problem for the population [11] because it is one of the reasons for cardiovascular diseases and type 2 diabetes [12]. A pooled estimate of MetS frequency in the general population based on International Diabetes Federation (IDF) criteria showed the following rates: 25% in Middle-East countries [13], 48% in Kingdom of Bahrain [14], 24.5% in Mainland China [15], 54% in Mexico [16], and 32.9% in Turkey [17].

It is important to control abdominal (including visceral) obesity, dyslipidemia, and increased glucose as components of the MetS already at the beginning of antipsychotic therapy. Visceral obesity occurs in 40–60% of schizophrenia patients and the accumulation of large amounts of abdominal and pericardial fat has considerable endocrine and cardiovascular consequences and negatively influences the health in general as well [18,19,20,21].

Patients with schizophrenia are at high risk of developing metabolic disturbances due to their predisposition to metabolism malfunctions, such as insulin resistance, which is then exacerbated by the subsequent use of antipsychotics. Changes in glucose and lipid metabolism are already observed after two weeks of treatment and reach a maximum after three months [22]. Baseline high-density lipoprotein (HDL)-cholesterol levels and waist circumference (WC) were identified as prognostic factors for the development of MetS in long-term antipsychotic therapy [23].

The contribution of thyroid-stimulating hormone (TSH) and thyroid hormones (THs) to the development of the metabolic disturbance has been assessed in only a few studies. THs play an important role in controlling energy homeostasis and can affect body composition. At the same time, insulin resistance is associated with low THs levels in non-diabetic individuals [24], and changes in body composition can affect THs levels. In general, schizophrenia patients have a lower level of free thyroxine (FT4) than that of healthy persons [25]. A strong negative correlation between negative symptoms according to the Positive and Negative Syndrome Scale (PANSS) scores and the level of TSH has been also detected [25]. Further, changes in THs levels are due to antipsychotic drug usage [26]. Serum-free triiodothyronine (FT3) levels exhibited positive associations with waist circumference (WC) and homeostasis model assessment of insulin resistance (HOMA-IR) and a negative association with body weight and body mass index (BMI) among both men and women in the general population of euthyroid subjects. Serum thyroid-stimulating hormone (TSH) levels showed a positive association with HOMA-IR in both men and women [27]. Baseline T4 and waist:hip ratio may be considered early markers of weight gain in second-generation antipsychotic treatment [28].

Western Siberia is a region characterized by low iodine content in the soil and plants due to the slightly acidic earth and low soil humus levels, permitting it to be, in terms of endemic goiter, referred to as a biogeochemical province. People who feed themselves with local plants and animals are at higher risk for endemic goiter, which accounts for 80% of thyroid diseases in the Tomsk region [29].

In this study, we aimed to investigate associations between body fat parameters, glucose and lipid profiles, TSH, and THs levels in Tomsk-region schizophrenia patients depending on the presence or absence of MetS. The novelty of the study is finding associations between body fat and biochemical parameters and to study the contribution of TSH and THs to the development of MetS in patients with schizophrenia, which is of particular importance for endemic regions for goiter. We hypothesize a possible positive association between THs levels and MetS in patients with schizophrenia.

## 2. Materials and Methods

Patients with schizophrenia were recruited from the inpatient departments of the Mental Health Research Institute, Tomsk National Research Medical Center of the Russian Academy of Sciences, and Tomsk Clinical Psychiatric Hospital and patients with MetS but without schizophrenia were recruited from the Hospital of the Siberian State Medical University. All hospitals mentioned are located in Tomsk, which is one of the Russian administrative Western Siberia centers where low iodine content in soil and plants is recorded. The study protocol was approved by the Local Bioethics Committee of the Mental Health Research Institute, Tomsk National Research Medical Center of the Russian Academy of Sciences (protocol N187, 24.04.2018). The study was carried out following the Code of Ethics of the World Medical Association (Declaration of Helsinki 1975, revised in Fortaleza, Brazil, 2013) for experiments involving humans, and all participants provided written informed consent.

### 2.1. Research Design

This was a comparative cross-sectional study of body fat and biochemical parameters in patients with schizophrenia treated with antipsychotics for at least 6 months carried out in 2018–2019. The study’s main outcome measures were fat parameters, glucose and lipid profiles, TSH, and THs levels with comparisons between the following groups:

1. Patients with schizophrenia without MetS.
2. Patients with schizophrenia and MetS.
3. Non-psychiatric general hospital inpatients with MetS.
4. Healthy controls to assess TSH and THs levels specifically for this region.

### 2.2. Study Population

We recruited 156 psychiatric inpatients of both sexes from January 2018 to December 2019. They met the following inclusion criteria:

1. A clinical diagnosis of schizophrenia according to the International Statistical Classification of Diseases and Related Health Problems, 10th Revision (ICD-10: F20).
2. Treatment with antipsychotics for at least 6 months before entering the study.
3. Age between 18 and 55 years.
4. Habitation in Western Siberia.

The exclusion criteria were as follows:

1. Migration throughout life.
2. A history of alcohol or drug abuse or eating disorders.
3. Presence of thyroid disease.
4. Undergoing medical treatments or research that might affect changes in metabolic parameters, TSH, and THs levels.

The diagnosis of schizophrenia was determined using the World Health Organization World Mental Health Composite International Diagnostic Interview (WHO WMH-CIDI). Patients with all schizophrenia subtypes were included in the study population. Symptom severity assessment was carried out with the Positive and Negative Syndrome Scale [30]. A total of 7 patients were not included in the study because they met the exclusion criterion number 1, and 22 patients were not included in the study because they met the exclusion criterion number 2.

We also recruited 35 inpatients from the general hospital of Siberian State Medical University with MetS (*n* = 35) of both sexes from January 2018 to December 2019 as a control group on body fat parameters, glucose and lipid profiles, TSH, and THs levels. They met the following inclusion criteria:

1. Presence of MetS according to the criteria of the International Diabetes Federation (IDF) [31].
2. Age between 18 and 55 years.
3. Habitation in Western Siberia.

The exclusion criteria were as follows:

1. Migration throughout life.
2. A history of alcohol or drug abuse, eating disorders, or any other psychiatric disorders.
3. Presence of thyroid disease.
4. Undergoing medical treatments or research that might affect changes in metabolic parameters, TSH, and THs levels.

Another control group included 35 healthy probands of both sexes, who voluntarily agreed to participate in the study. They met the following inclusion criteria:

1. Age between 18 and 55 years.
2. Habitation in Western Siberia.

The exclusion criteria were as follows:

1. Migration throughout life.
2. A history of MetS, thyroid disease, alcohol or drug abuse, eating disorders, schizophrenia, and other mental disorders.
3. Undergoing medical treatments or research that might affect changes in metabolic parameters, TSH, and THs levels.

### 2.3. MetS Definition

We used the criteria of the IDF. These criteria [31] demand that MetS is diagnosed in a patient with central obesity (WC more than 94 cm in men and more than 80 cm in women) and the presence of any two of the following four signs:

1. The concentration of triglycerides in serum is higher than 1.7 mmol/L (150 mg/dL) or lipid-lowering therapy is carried out.
2. The concentration of high-density lipoprotein in serum is below 1.03 mmol/L (40 mg/dL) in men and 1.29 mmol/L (50 mg/dL) in women.
3. The arterial blood pressure level is systolic above 130 mmHg or diastolic above 85 mmHg (or with the treatment of previously diagnosed hypertension).
4. Serum glucose concentration is greater than 5.6 mmol/L (100 mg/dL) (or previously diagnosed type 2 diabetes).

### 2.4. Body Fat Composition Assessment

WC was defined with a measuring tape. The anthropometric measurements below fully covered the composition of the entire body fat compartment, including indicators of both subcutaneous and visceral fat.

The body fat percentage, visceral fat level, body weight, and BMI were measured through the non-invasive bioimpedance analysis with an “Omron BF508” scale and body composition monitor. The percentage body fat data was checked with body fat ranges reported by Gallagher et al. [32] for different ages in white people. Indicators of subcutaneous fat (total fat fold) were determined using an electronic caliper. The total fat fold represents the sum of the shoulder, back, abdomen, and lower-leg fat folds. The method of non-invasive bioimpedance analysis yields objective data on the composition of the body fat component and can be easily reproduced including in routine psychiatric and other clinical practice.

### 2.5. Blood Sampling

Blood samples were drawn after 12 h overnight fasting by antecubital venipuncture into tubes (BD Vacutainer) with a clot activator (CAT). To isolate serum samples, the tubes were centrifuged for 30 min at 2000× *g* at 4 °C. The serum was stored at −20 °C (or −80 °C), until analysis.

### 2.6. Biochemical Parameters Assessment

The concentration of total cholesterol, high-density lipoproteins, triglycerides, and glucose in blood serum was determined by colorimetric enzymatic methods applying standard commercial kits (Cormay, Poland). Concentrations of low density and very-low-density lipoproteins were calculated from the formula of Friedewald et al. [33]. The atherogenic index was calculated from the formula offered by Klimov [34].

### 2.7. TSH and THs Levels Assessment

The serum levels of free triiodothyronine (FT3), FT4, and TSH in patients with schizophrenia and controls were identified by using the kits for enzyme-linked immunosorbent assay (ELISA, AO Vector-Best, Novosibirsk, Russia; standard—EN ISO 13485 (certified by medical device certification GmbH, Berlin, Germany)). Normal values were 4.0–8.6 pmol/L, 10.3–24.5 pmol/L, and 0.4–4.0 mME/L, respectively.

### 2.8. Statistical Analysis

Statistical analyses were performed using the SPSS software for Windows, version 23.0, as well as using the scripting programming language R 3.6.1 in RStudio 1.2.5001. To calculate sample size, we used specialized software complex IBM SPSS Sample power. In our study, there were 4 groups. The key parameters were connected with hormonal status, and we use these continuous variables to calculate the sample size. For 90% power, the minimum sample size was 32. The Shapiro–Wilk test was used to analyze the normal distribution of the variables (*p* > 0.05). The statistical significance of the differences was evaluated using the Mann–Whitney *U*-test for independent samples with Bonferroni correction for multiple comparisons. Categorical variables were analyzed using the chi-squared test. Correlation analysis was performed using the Spearman’s rank correlation coefficient. Multiple regression analyses were performed considering FT3, FT4, and TSH as dependent variables and statistically significant correlated metabolic parameters as an independent variable. Descriptive statistics were shown as median (Me) with 25% and 75% quartiles (Q1; Q3). *A p*-value of less than 0.05 was considered significant.

## 3. Results

### 3.1. Demographics and Baseline Parameters of Study Groups

A total of 156 patients with schizophrenia were divided into two groups depending on MetS presence (*n* = 56) or absence (*n* = 100). The other two groups included general hospital inpatients with MetS but without schizophrenia (*n* = 35) and healthy probands (*n* = 35).

No significant statistical differences existed between these groups concerning age and sex composition except for general hospital inpatients with MetS who had a significantly higher median age in comparison to that of both groups’ with schizophrenia (Table 1). Differences in any parameters between the general hospital inpatients with MetS and healthy people were not evaluated as this was not the subject of the study.

### 3.2. TSH and THs Levels

TSH and THs levels were normal in all compared groups. Patients with schizophrenia and MetS had significantly higher levels of FT3 and FT4 compared with those of schizophrenia patients without MetS, but their level was lower compared with those of general hospital inpatients with MetS. Furthermore, TSH and THs levels were lower in patients with schizophrenia regardless of the MetS compared with those of healthy probands (Table 2).

### 3.3. Body Fat Composition

As was expected, schizophrenia patients with MetS had a significantly higher body weight, WC, body fat percentage result, and total fat fold than patients with schizophrenia without MetS, at the same time, the people with schizophrenia without MetS had had lower body fat composition all parameters than general hospital inpatients with MetS (Table 3).

### 3.4. Glucose and Lipid Profiles

We found significant statistical differences in glucose levels between all groups. Patients with schizophrenia and MetS had significantly higher levels than patients without MetS did. Glucose levels in general hospital inpatients with MetS were higher in comparison to that in both groups’ schizophrenia patients (Table 4)

We did not observe significant differences in total cholesterol and low-density lipoproteins between groups. Schizophrenia patients with MetS had significantly higher levels of triglycerides in comparison to those in the other groups and very-low-density lipoproteins in comparison to the schizophrenia patients without MetS, and low indicators of high-density lipoproteins and higher levels of the atherogenic index than those in patients from other groups (Table 4).

### 3.5. The Correlation between Body Fat Parameters, Glucose and Lipid Profiles, TSH, and THs Levels

**Figure 1:**
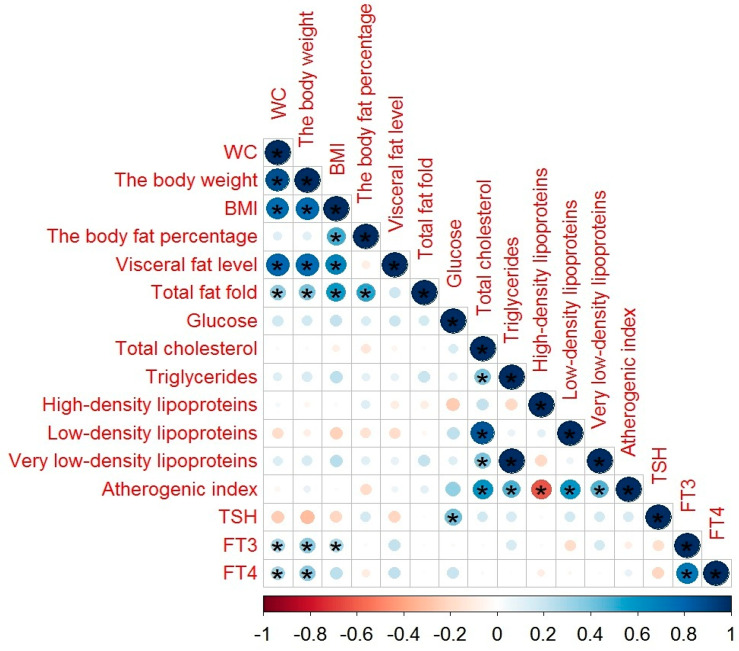
The correlation between body fat parameters, glucose and lipid profiles, TSH, and THs levels in schizophrenia patients with MetS: in this figure, red and blue circles mean negative and positive correlations, respectively; the size and color intensity of circles are proportional to the correlation coefficient; * *p*-value < 0.05; in the bottom, the legend of color intensity shows the rate of correlations and the corresponding relations.

The correlations between body fat parameters, glucose and lipid profiles, TSH, and THs levels in schizophrenia patients with MetS are illustrated in Figure 1. TSH showed a moderate correlation with glucose (*r* = 0.405, *p* = 0.002); FT3 had a moderate correlation with body weight (*r* = 0.413, *p* = 0.002) and weak correlation with WC (*r* = 0.328, *p* = 0.013) and BMI (*r* = 0.295, *p* = 0.03); FT4 showed a weak correlation with WC (*r* = 0.287, *p* = 0.032) and body weight (*r* = 0.374, *p* = 0.005).

**Figure 2:**
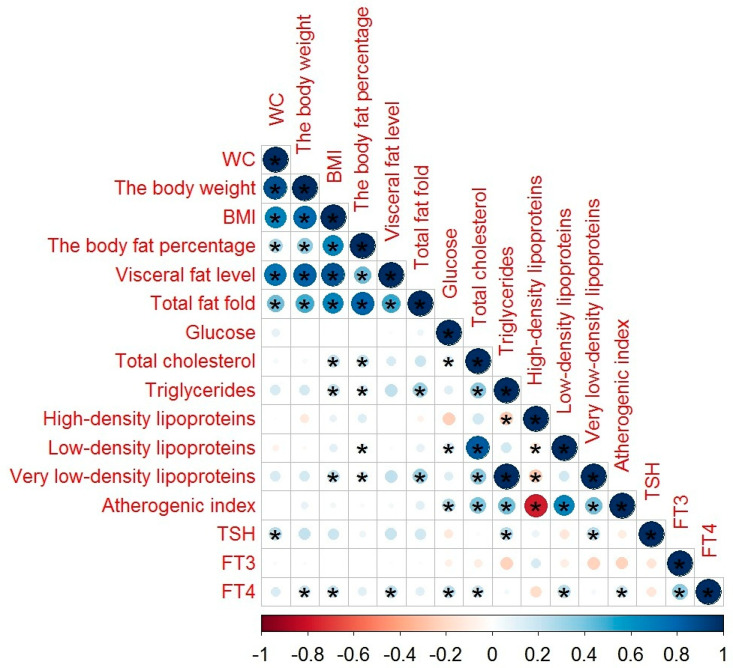
The correlation between body fat parameters, glucose and lipid profiles, TSH, and THs levels in schizophrenia patients without MetS: in this figure, red and blue circles mean negative and positive correlations, respectively; the size and color intensity of circles are proportional to the correlation coefficient; * *p*-value < 0.05; in the bottom, the legend of color intensity shows the rate of correlations and the corresponding relations.

The correlations between body fat parameters, glucose and lipid profiles, and THs levels in schizophrenia patients without MetS are illustrated in Figure 2. TSH showed a weak correlation with WC (*r* = 0.222, *p* = 0.031), triglycerides (*r* = 0.262, *p* = 0.011), and very low-density lipoproteins (*r* = 0.262, *p* = 0.011); FT4 had a weak correlation with BMI (*r* = 0.249, *p* = 0.014), body weight (*r* = 0.214, *p* = 0.037), visceral fat level (*r* = 0.245, *p* = 0.016), glucose (*r* = 0.345, *p* = 0.001), total cholesterol (*r* = 0.206, *p* = 0.043), low-density lipoproteins (*r* = 0.249, *p* = 0.014), and atherogenic index (*r* = 0.216, *p* = 0.033).

**Figure 3:**
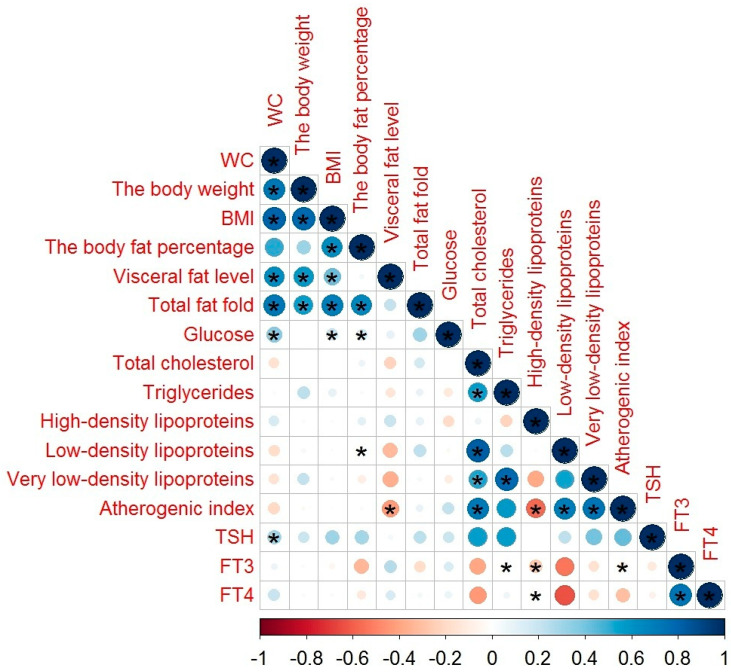
The correlation between body fat parameters, glucose and lipid profiles, TSH, and THs levels in general hospital inpatients with MetS: in this figure, red and blue circles mean negative and positive correlations, respectively; the size and color intensity of circles are proportional to the correlation coefficient; * *p*-value < 0.05; in the bottom, the legend of color intensity shows the rate of correlations and the corresponding relations.

The correlations between body fat parameters, glucose and lipid profiles, TSH, and THs levels in general hospital inpatients with MetS are illustrated in Figure 3. TSH showed a weak correlation with WC (*r* = 0.386, *p* = 0.020); FT3 had a moderate negative correlation with high-density lipoproteins (*r* = −0.578, *p* = 0.001), weak negative correlation with atherogenic index (*r* = −0.353, *p* = 0.037), and very weak negative correlation with triglycerides (*r* = −0.039, *p* = 0.043); FT4 showed a very weak correlation with high-density lipoproteins (*r* = 0.037, *p* = 0.015).

We then performed a multiple regression analysis, considering THs as a dependent variable and statistically significant correlated metabolic parameters as an independent variable. We found TSH maintained an association only with sex (adjusted *R*^2^ = 0.038, β = 0.213, *p* = 0.005, data not shown). FT3 maintained an association with MetS, sex, age, and high-density lipoproteins (Table 5).

FT4 maintained an association with MetS, sex, age, and glucose and tended to associate with WC and visceral fat level (Table 6).

## 4. Discussion

The present study examined body fat parameters, glucose and lipid profiles, TSH, and THs levels in patients with an ICD-10 diagnosis of schizophrenia without (*n* = 56) or with (*n* = 100) MetS in comparison to two control groups of nonpsychiatric inpatients (*n* = 35) with MetS and healthy persons (*n* = 35). Patients with schizophrenia were recruited after treatment with antipsychotics for at least six months before entering the study, which was carried out in Western Siberia—a biogeochemical region with endemic goiter.

The most important findings in the present study are as follows:

1. Patients with schizophrenia and MetS had significantly higher levels of FT3 and FT4 pmol/L compared with those in schizophrenia patients without MetS.
2. Schizophrenia patients with MetS had significantly higher triglyceride levels and atherogenic indices and lower parameters of high-density lipoproteins and glucose in comparison to those in general hospital inpatients with MetS.
3. FT3 maintained an association with MetS, sex, age, and high-density lipoproteins. FT4 maintained an association with MetS, sex, age, and glucose and tended to associate with WC.

### 4.1. Demographics and Baseline Parameters

General hospital inpatients with MetS had a significantly higher age (Table 1) in comparison with that in both groups of schizophrenia patients (*p* = 0.0001 in either comparison). Considering that patients were included in the study at the same time in parallel, which was determined by the study design, it can be assumed that the higher age of general hospital inpatients with MetS is explained by the common pattern of MetS development in the general population. In particular, the overall prevalence of MetS increases with age—15–39 years: 13.9%; 40–59 years: 26.4%; 60 years: 32.4% [15]. In contrast to the pattern observed in the general population, where the prevalence of MetS is low in young people and increases with age, its prevalence among patients with schizophrenia is significantly higher in younger age groups [35]. Although the prevalence of MetS increases with age and an age over 40 years old has been recognized as a significant risk factor for MetS [36], its prevalence with increasing age in patients with schizophrenia is lower than that in the general population.

### 4.2. TSH and THs Levels

TSH, FT3, and FT4 levels were consistent with the norm in all compared groups (Table 2). We assume that the obtained results are due to the structure of food products in Western Siberia that have changed in recent decades toward not only local food but also products from other regions, thanks to market reforms in Russia. The data obtained suggest that THs levels differences in patients with schizophrenia compared with those in other groups may be associated with the influence of antipsychotic therapy—the influence of schizophrenia itself also cannot be ruled out [37]. Patients with schizophrenia and MetS had significantly higher THs levels compared with schizophrenia patients without MetS and general hospital inpatients with MetS. General hospital inpatients with MetS had the lowest TSH and the highest FT4 compared to both groups of patients with schizophrenia. Possible explanations for these results are the effects of psychotropic therapy received by patients with schizophrenia [38], and the higher age of general hospital inpatients with MetS [39], respectively. Li et al. [28] showed a relationship between increased FT4 levels and a higher prevalence of type 2 diabetes in both men and women, which is important concerning MetS. A previous study showed signs of subclinical hypothyroidism in patients with chronic schizophrenia [25], which contributes to an increase in cholesterol and blood pressure [40]. Associations were found between thyroid dysfunction and cardiovascular risk factors, and the development of MetS [41], as well as between TSH levels and the presence of MetS in patients with schizophrenia [42], and similarly in the general population [43]; therefore, it is necessary to monitor the levels of TSH and THs in patients with schizophrenia as possible risk factors for developing MetS.

### 4.3. Body Fat Composition

Patients with schizophrenia and MetS had a significantly larger fat component, including both visceral and subcutaneous fat, compared with patients with schizophrenia without MetS (Table 3) which is expected, considering that abdominal obesity is the main diagnostic criterion for MetS by IDF criteria. Previous studies have shown that patients with chronic schizophrenia, even with normal body weight, have higher levels of visceral fat than healthy people do [18]; therefore, it might be suggested that monitoring the fat component in patients with schizophrenia treated with antipsychotics is an essential part of all management tactics for patients with this severe mental disorder to prevent the development of MetS [44].

BMI and visceral fat in general hospital inpatients with MetS was significantly higher than that in both groups of patients with schizophrenia. This may be related to their older age [45], and, consequently, to the longer duration of obesity, the risk of developing insulin resistance, and a pre-diabetic state.

### 4.4. Glucose and Lipid Profiles

Schizophrenia patients with MetS had a higher level of triglycerides, HDL, and atherogenic index than that in general hospital inpatients with MetS who had higher glucose levels (Table 4). These disturbances would increase the risk of developing type 2 diabetes and coronary heart disease. Mitchel et al. [46], in a meta-analysis, demonstrated an increased influence of lipid metabolism on the development of MetS. They found a decrease in HDL in 42.6% of patients, hypertriglyceridemia in 39.3% of patients, hypertension in 38.7% of patients, and hyperglycemia in 18.8% of patients [46]. It can be assumed that there is another pathophysiological mechanism for the development of MetS in mental illness, which antipsychotic therapy and the psychopathological process may contribute to.

### 4.5. The Correlation between Body Fat Parameters, Glucose and Lipid Profiles, TSH, and THs Levels

A correlation of HDL levels with THs levels (Figure 1, Figure 2 and Figure 3) confirmed the results of the study of Huang et al. [47]. FT3 (Table 5) may alter lipid metabolism by acting on the mRNA of cholesterol 7a-hydroxylase, scavenger receptor-BI protein, or ATP-binding cassette transporter 1 [47]. FT4 concentrations (Table 6) showed a negative association with WC. These results are in line with the results of De Pergola [48], demonstrating a negative relationship between the WC, BMI, and FT4 levels. The association of FT4 and glucose obtained in our study confirms the participation of THs in the regulation of carbohydrate metabolism. A possible mechanism of action is mediated at the level of deiodinase receptors [49].

### 4.6. Strengths and Limitations

The main limitation of the present study is the comparison of body fat parameters, glucose and lipid profiles, and THs levels between groups of patients who had significant age differences (general hospital inpatients with MetS had a significantly higher average age in comparison with that of both groups of schizophrenia patients, Table 1); however, this fact reflects clinical reality and demonstrates the increased vulnerability of patients with schizophrenia to the development of MetS at a younger age. This is due, among other things, to the influence of one of the main iatrogenic factors in the form of antipsychotic treatment [50,51], which patients with schizophrenia take for a long time, and antipsychotic therapy remains one of the important and necessary factors in the intervention for this mental disorder. The average age of patients in each compared group was also in the same age cohort (40–59 years) for measuring the percentage of body fat according to data obtained in a study of different ethnic groups [32], which partially eliminates the specified limitation. Another limitation is the absence of patient stratification according sex. Strich et al. [52] showed reliable data on the characteristics of the hormonal level depending on age and sex. FT3 decreases throughout life and is significantly higher among women. Its alignment between the sexes occurs with age. FT4 is reduced to a lesser extent, but also more among women than among men. Among older people, women have higher FT4 levels. In contrast, TSH decreases up to 50 years and then increases slightly in both sexes. The considerable strengths of our study include the representativeness of this sample for typical cases in routine psychiatric practice; the unique sample recruited in Western Siberia, which is a biogeochemical region of endemic goiter; analysis of associations between body fat and biochemical parameters play a role in MetS in patients with schizophrenia.

## 5. Conclusions

In summary, our findings showed some peculiarities of body fat parameters, glucose and lipid profiles, TSH, and THs levels in MetS in patients with schizophrenia who take antipsychotic therapy. Seeking MetS parameters in a region characterized by low iodine content in soils and plants due to slightly sour soils and low soil humus levels, permitting it to be, in terms of endemic goiter, referred to as a biogeochemical province will help us better recognize and treat MetS in patients with schizophrenia in similar regions. Future investigations on this topic, including prospective studies, could lead to the development of personalized health care to this group of psychiatric patients with somatic complications. Meanwhile, the question of whether thyroid dysfunction can be considered a risk of MetS in patients with schizophrenia or whether MetS itself leads to it while taking antipsychotics is still unanswered. Furthermore, the influence of schizophrenia on both the formation of MetS and thyroid dysfunction cannot be ignored.

## Figures and Tables

**Table 1:** Demographics and baseline parameters of study groups.

Parameter	Patients with Schizophrenia without MetS (*n* = 100)	Patients with Schizophrenia and MetS (*n* = 56)	General Hospital Inpatients with MetS (*n* = 35)	Healthy Probands (*n* = 35)	*p*-Value
Age, years	42 [36; 51]	45.5 [35.5; 54]	57 [49; 60]	44 [39; 48.5]	*p*_1–2_ = 0.480 *p*_1–3_ = 0.0001 * p_1-4_ = 0.759 *p*_2–3_ = 0.0001 * p_2–4_ = 0.552
Sex (Male, *n* (%)/Female, *n* (%))	45 (45.0)/55 (55.0)	23 (41.1)/33 (58.9)	20 (57.1)/15 (42.9)	10 (28.6)/25 (71.4)	*p*_1–2_ = 0.737 *p*_1–3_ = 0.242 *p*_1–4_ = 0.111 *p*_2–3_ = 0.195 *p*_2–4_ = 0.267
Duration of disorder, years	14.5 [8; 22.5]	16 [10; 22]	NA	NA	*p*_1–2_ = 0.285
Schizophrenia onset age	27 [20; 35]	26 [20; 32.5]	NA	NA	*p*_1–2_ = 0.994
PANSS, total score	107 [96.5; 116]	103.5 [93.5; 111]	NA	NA	*p*_1–2_ = 0.084

* *p* < 0.05—statistically significant difference; comparisons between groups were performed using the chi-squared test for sex and Mann–Whitney *U*-test for independent samples with Bonferroni correction for multiple comparisons for the rest of the variables; categorical variables were analyzed using Me [Q1; Q3]—median and quartiles (first and third); *p*_1–2_—the significance level of differences when comparing the parameters of patients with schizophrenia with metabolic syndrome (MetS) and without MetS, *p*_1–3_—the significance level of differences when comparing parameters of patients with schizophrenia without MetS and general hospital inpatients with MetS, *p*_1–4_—the significance level of differences when comparing the parameters of healthy people and patients with schizophrenia without MetS, *p*_2–3_—the significance level of differences when comparing indicators of patients with schizophrenia and MetS and general hospital inpatients with MetS, *p*_2–4_—the significance level of differences when comparing the parameters of healthy people and patients with schizophrenia and MetS. PANSS—Positive and Negative Syndrome Scale; NA—not applicable.

**Table 2:** Thyroid-stimulating hormone (TSH) and thyroid hormones (THs) levels.

Parameter	Patients with Schizophrenia without MetS (*n* = 100)	Patients with Schizophrenia and MetS (*n* = 56)	General Hospital Inpatients with MetS (*n* = 35)	Healthy Probands (*n* = 35)	*p*-Value
TSH, mME/L	1.38 [0.81; 2.03]	1.58 [0.83; 2.58]	1.03 [0.72; 1.40]	3.04 [2.09; 4.78]	*p*_1–2_ = 0.293 *p*_1–3_ = 0.092 *p*_1–4_ = 0.0001 * p_2–3_ = 0.172 *p*_2–4_ = 0.0001 *
FT3, pmol/L	3.24 [2.81; 3.66]	3.68 [3.25; 5.50]	5.85 [3.84; 7.09]	6.28 [5.83; 7.03]	*p*_1–2_ = 0.0001 * p_1–3_ = 0.0001 * p_1–4_ = 0.0001 * p_2–3_ = 0.024 * p_2–4_ = 0.0001 *
FT4, pmol/L	10.81 [9.76; 12.3]	12.68 [10.73; 15.54]	16.39 [14.16; 18.75]	15.4 [13.73; 17.66]	*p*_1–2_ = 0.0001 * p_1–3_ = 0.0001 * p_1–4_ = 0.0001 * p_2–3_ = 0.0001 * p_2–4_ = 0.0001 *

* *p* < 0.05—statistically significant difference; comparisons between groups were performed using the Mann–Whitney *U*-test for independent samples with Bonferroni correction for multiple comparisons; categorical variables were analyzed using Me [Q1; Q3]—median and quartiles (first and third); *p*_1–2_—the significance level of differences when comparing the parameters of patients with schizophrenia and MetS and without MetS; *p*_1–3_—the significance level of differences when comparing parameters of patients with schizophrenia without MetS and general hospital inpatients with MetS; *p*_1–4_—the significance level of differences when comparing the parameters of healthy people and patients with schizophrenia without MetS; *p*_2–3_—the significance level of differences when comparing indicators of patients with schizophrenia and MetS and general hospital inpatients with MetS; *p*_2–4_—the significance level of differences when comparing the parameters of healthy people and patients with schizophrenia and MetS. FT3—free triiodothyronine; FT4—thyroxine.

**Table 3:** Body fat composition parameters (Me [Q1; Q3]).

Parameter	Patients with Schizophrenia without MetS (*n* = 100)	Patients with Schizophrenia and MetS (*n* = 56)	General Hospital Inpatients with MetS (*n* = 35)	*p*-Value
Body weight, kg	68.5 [60.4; 75.9]	90.3 [77.1; 105.2]	94.4 [86.6; 103.8]	*p*_1–2_ = 0.0001 * p_1–3_ = 0.0001 * p_2–3_ = 0.132
WC, cm	83 [87; 91]	103.5 [95; 114]	105 [99; 112.5]	*p*_1–2_ = 0.0001 * p_1–3_ = 0.0001 * p_2–3_ = 0.202
BMI	23.9 [21.2; 26.4]	30.6 [26.9; 35]	35.1 [31.5; 40]	*p*_1–2_ = 0.0001 * p_1–3_ = 0.0001 * p_2–3_ = 0.0001 *
Body fat percentage result	29.7 [21.9; 37.6]	39.9 [34.2; 47.3]	44.8 [38.5; 50.9]	*p*_1–2_ = 0.0001* p_1–3_ = 0.0001 * p_2–3_ = 0.863
Visceral fat level	6 [4; 8]	10 [8; 13]	14 [11; 16]	*p*_1–2_ = 0.0001 * p_1–3_ = 0.0001 * p_2–3_ = 0.0001 *
Total fat fold, mm	66 [49; 91]	116 [91; 133]	122 [93.5; 146.5]	*p*_1–2_ = 0.0001 * p_1–3_ = 0.0001 * p_2–3_ = 0.388

* *p* < 0.05—statistically significant difference; comparisons between groups were performed using the Mann–Whitney *U*-test for independent samples with Bonferroni correction for multiple comparisons; categorical variables were analyzed using Me [Q1; Q3]—median and quartiles (first and third); *p*_1–2_—the significance level of differences when comparing the parameters of patients with schizophrenia and MetS and without MetS; *p*_1–3_—the significance level of differences when comparing parameters of patients with schizophrenia without MetS and general hospital inpatients with MetS; *p*_2–3_—the significance level of differences when comparing indicators of patients with schizophrenia and MetS and general hospital inpatients with MetS. BMI—body mass index.

**Table 4:** Glucose and lipid profiles (Me [Q1; Q3]).

Parameter	Patients with Schizophrenia without MetS (*n* = 100)	Patients with Schizophrenia and MetS (*n* = 56)	General Hospital Inpatients with MetS (*n* = 35)	*p*-Value
Glucose, mmol/L	4.59 [4.17; 5.1]	5.2 [4.7; 5.88]	5.4 [5.1; 6.45]	*p*_1–2_ = 0.0001 * p_1–3_ = 0.0001 * p_2–3_ = 0.018 *
Total cholesterol, mmol/L	4.53 [4.0; 5.2]	4.45 [3.95; 5.4]	5.62 [3.95; 5.49]	*p*_1–2_ = 0.998 *p*_1–3_ = 0.595 *p*_2–3_ = 0.613
Triglycerides, mmol/L	1.1 [0.77; 1.42]	2.0 [1.59; 2.5]	1.8 [1.37; 2.09]	*p*_1–2_ = 0.0001 * p_1–3_ = 0.0001 * p_2–3_ = 0.028 *
High-density lipoproteins, mmol/L	1.08 [0.9; 1.28]	0.81 [0.7; 1.0]	1.04 [0.9; 1.3]	*p*_1–2_ = 0.0001 * p_1–3_ = 0.739 *p*_2–3_ = 0.0001 *
Low-density lipoproteins, mmol/L	2.93 [2.49; 3.34]	2.67 [2.06; 3.61]	2.9 [2.13; 3.82]	*p*_1–2_ = 0.253 *p*_1–3_ = 0.992 *p*_2–3_ = 0.453
Very-low-density lipoproteins, mmol/L	0.5 [0.35; 0.65]	0.9 [0.73; 1.14]	0.85 [0.68; 0.96]	*p*_1–2_ = 0.0001 * p_1–3_ = 0.0001 * p_2–3_ = 0.148
Atherogenic index	3.26 [2.44; 4.71]	4,34 [3.35; 6.1]	3.35 [2.35; 4.42]	*p*_1–2_ = 0.0001 * p_1–3_ = 0.973 *p*_2–3_ = 0.012 *

* *p* < 0.05—statistically significant difference; comparisons between groups were performed using the Mann–Whitney *U*-test for independent samples with Bonferroni correction for multiple comparisons; categorical variables were analyzed using Me [Q1; Q3]—median and quartiles (first and third); *p*_1–2_—the significance level of differences when comparing the parameters of patients with schizophrenia and MetS and without MetS; *p*_1–3_—the significance level of differences when comparing parameters of patients with schizophrenia without MetS and general hospital inpatients with MetS; *p*_2–3_—the significance level of differences when comparing indicators of patients with schizophrenia and MetS and general hospital inpatients with MetS.

**Table 5:** Determinants of free triiodothyronine (FT3) concentrations in multiple regression analysis.

Variable	Β	*p*-Value
Age, years	−0.150	0.022 *
Sex	−0.234	0.0001 *
MetS	0.541	0.0001 *
WC, cm	−0.086	0.569
BMI	0.143	0.294
Body weight, kg	0.022	0.898
Triglycerides, mmol/L	0.003	0.966
High-density lipoproteins, mmol/L	−0.172	0.033 *
Atherogenic index	−0.074	0.416

* *p* < 0.05—statistically significant difference; adjusted *R*^2^ = 0.385.

**Table 6:** Determinants of free thyroxine (FT4) concentrations in multiple regression analysis.

Variable	Β	*p*-Value
Age, years	−0.169	0.014 *
Sex	−0.211	0.001 *
MetS	0.433	0.0001 *
WC, cm	−0.289	0.068
BMI	−0.037	0.791
Body weight, kg	0.175	0.352
Visceral fat level	0.232	0.094
Glucose, mmol/L	0.192	0.009*
Total cholesterol, mmol/L	0.209	0.289
High-density lipoproteins, mmol/L	−0.201	0.164
Low-density lipoproteins, mmol/L	−0.086	0.596
Atherogenic index	−0.153	0.289

* *p* < 0.05—statistically significant difference; adjusted *R*^2^ = 0.388.
